# Case report: Diagnostic challenges of papulonodular mucinosis in a 67-year-old female

**DOI:** 10.1177/2050313X241311372

**Published:** 2025-01-09

**Authors:** Holly E Zahary Loreman, Karen I Holfeld

**Affiliations:** 1 University of Calgary Cumming School of Medicine, Calgary, AB, Canada; 2 Division of Dermatology, University of Saskatchewan College of Medicine, Saskatoon, SK, Canada

**Keywords:** Mucinosis, papulonodular mucinosis, papular and nodular mucinosis of Gold, cutaneous lupus mucinosis, lupus erythematosus, dermatopathology, hydroxychloroquine

## Abstract

Papulonodular mucinosis is a rare dermatological condition characterized by mucin deposition in the dermis, leading to the formation of papules and nodules that can occur with, or antedate, autoimmune connective tissue diseases. This case report presents a 67-year-old female with a chronic history of cutaneous mucinosis, which posed significant diagnostic challenges. Despite various treatments and extensive diagnostic workup, her condition evolved, highlighting the difficulties in diagnosing papulonodular mucinosis, especially in the absence of systemic lupus erythematosus and antinuclear antibody positivity. The unusual presentation and diagnostic complexity underscore the need for awareness and thorough investigation in similar cases.

## Introduction

Cutaneous mucinoses are a broad category of disorders characterized by abnormal deposition of mucin in the skin producing diffuse or focal lesions. The etiology of the increased mucin accumulation is unclear, although it has been theorized that elevated serum immunoglobulins, autoantibodies, or cytokines such as IL-1, TNF, and TGF-β may play a role.^1^

Papulonodular mucinosis (PNM) is a subtype of cutaneous mucinosis associated with autoimmune disease. It is commonly associated with lupus erythematosus (LE) and more rarely has been seen in dermatomyositis and systemic sclerosis. When associated with LE, alternative terms include cutaneous lupus mucinosis, papular and nodular mucinosis of lupus, and papular and nodular mucinosis of Gold. PNM occurs in approximately 1%–2% of patients with LE.^1^ Although it occurs most commonly in patients with an established LE diagnosis, it can also antedate the disease. Most patients who develop PNM have systemic lupus erythematosus (SLE), with the minority having isolated cutaneous LE. The lesions are typically asymptomatic (although pruritic lesions have been described)^2^ flesh-colored 0.5–2 cm papules and nodules, commonly distributed on the back, chest, and upper extremities.^3^

Histologically, abundant mucin deposition and fibroblast proliferation is seen within the dermis, occasionally with superficial perivascular lymphocytic infiltrate.^1,4^ Increased collagen bundles are often found in the papillary and mid-reticular dermis, with mucin accumulation between the bundles. There may be linear or granular immunoglobulins (IgG/IgM) and/or C3 at the basement membrane,^4,5^ while the epidermis is relatively normal. Although mucin deposits in the superficial dermis are seen in lupus, other typical histologic findings of cutaneous LE, such as epidermal and dermal inflammation and vacuolar degeneration at the epidermal basal layer, are typically absent in PNM.^5^

Treatment of PNM is comparable to that of LE, including sun protection, corticosteroids (topical, oral, or intralesional), and oral antimalarials, although response to treatment is variable.^1,3,4,5^ Successful treatment with retinoids, cyclophosphamide, methotrexate, plasmapheresis, dermabrasion, laser, excision, or intralesional hyaluronidase has also been described.^6,7^

Diagnosing this rare condition is challenging, particularly when it occurs without an associated SLE diagnosis. This report details the diagnostic journey of a 67-year-old female with antinuclear antibody (ANA)-negative cutaneous mucinosis in the absence of SLE, emphasizing the importance of comprehensive evaluation in atypical presentations.

## Case report

A 67-year-old female was referred to dermatology with a four-year history of pruritic, subcutaneous papules and nodules on the abdomen and back. Her past medical history was noncontributory. Previous treatments included topical clobetasol, narrowband UVB, and isotretinoin, which provided limited relief. Upon initial presentation to dermatology, she was diagnosed with nodular lichen myxedematosus and treated with methotrexate (MTX). Despite a period of stability, she continued to experience pruritus and developed new nodules, including involvement of her forehead. After approximately 2 years, MTX was discontinued due to worsening of her symptoms and aspartate transaminase (AST) elevation. Following discontinuation, she developed worsening eruptions to her trunk and extremities, and her pruritus intensified.

**Figure 1.**
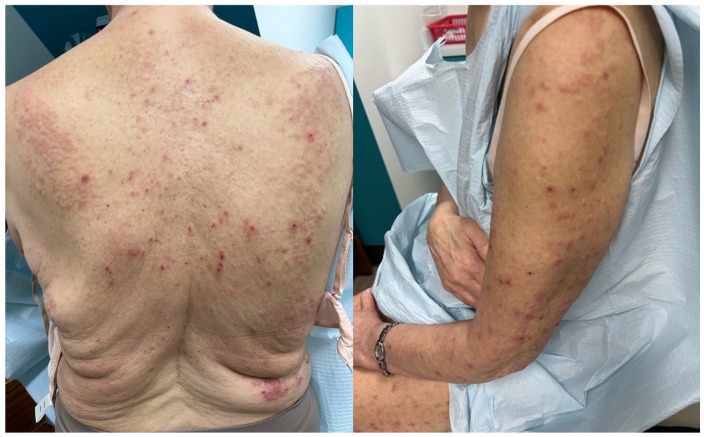
Diffuse flesh-colored and erythematous excoriated papules and nodules on the back and upper extremity after discontinuing methotrexate treatment.

Physical examination revealed dozens of flesh-colored to erythematous excoriated papules and subcutaneous nodules involving the abdomen, back, and eventually the upper and lower extremities (Figure 1). Her forehead lesions presented with erythematous papules and right-sided eyebrow hair loss. There were no signs of any systemic involvement of LE.

**Figure 2.**
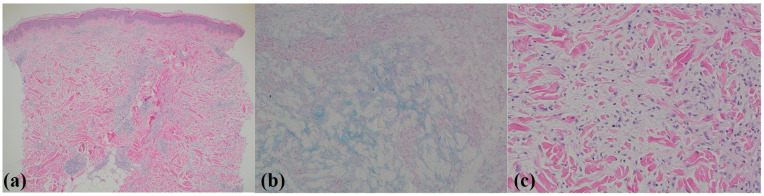
A skin biopsy showing (a) unremarkable epidermis, superficial and deep perivascular and interstitial lymphohistiocytic infiltrate with rare eosinophils within the dermis, 4× magnification. (b) Colloidal iron stain highlighting dermal mucin, perivascular and interstitial lymphohistiocytic infiltrate within the mid-reticular dermis, 10× magnification. (c) Increased number of fibroblasts within the collagen bundles and interstitial mucin, 20× magnification.

The patient underwent multiple skin punch biopsies for diagnostic clarification. Repeated trunk and extremity biopsies revealed similar findings of superficial and deep perivascular and interstitial lymphohistiocytic infiltrate (Figure 2(a)), and increased fibroblasts and collagen bundles with interstitial dermal mucin deposition confirmed with colloidal iron stain (Figure 2(b) and (c)). The differential diagnosis included PNM, lichen myxedematosus, and scleromyxedema. Given the distinct clinical features of these conditions, further investigation into PNM was conducted, which was clinically correlated with her condition.

An additional biopsy was obtained of her forehead lesions, which flared prior to methotrexate discontinuation. This demonstrated perivascular and periadnexal lymphohistiocytic infiltrate with poorly formed granulomas, mildly increased mucin, and scattered T-cells in the dermis. The case was presented at provincial dermatopathology rounds, with consensus that the differential diagnosis included granulomatous rosacea, infectious etiology (despite negative microorganism stains), sarcoidosis, and connective tissue disease. The relationship of this new eruption to her original condition remains unclear.

Other investigations included monitoring bloodwork and urinalysis every 2 months while on MTX, which were unremarkable aside from an elevated AST of 46 U/L (5–35 U/L) eighteen months after starting MTX. Initial lab tests included TSH, SPEP, immunoglobulin panel, and ANA titres which were within normal limits. There are no specific antibodies associated with PNM; however, since most patients with PNM have SLE and are ANA positive, her ANA titres were tested repeatedly to assess for lupus. Her titres were initially negative, but she did eventually have a borderline positive result at 1:80.

**Figure 3.**
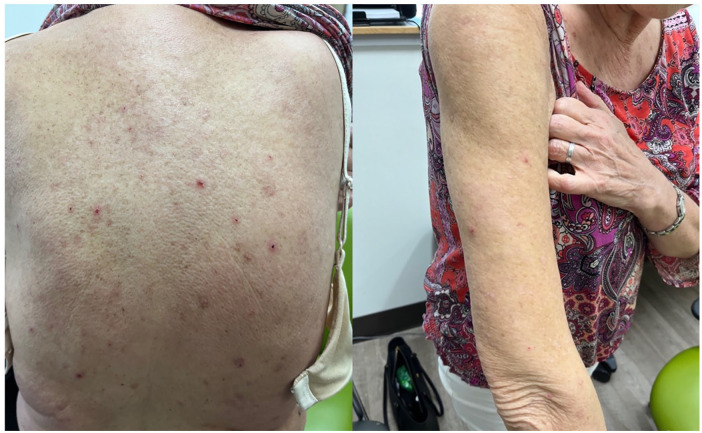
Scant erythematous excoriated papules and nodules on the back and upper extremity after 3 months of treatment with hydroxychloroquine.

After MTX discontinuation, the patient was treated with hydroxychloroquine 300 mg QD (later increased to 400 mg QD) and betamethasone 0.1% cream. After 3 months of treatment, she reported a reduction in pruritus, decrease in the formation of new papules/nodules, and gradual resolution of existing lesions (Figure 3).

## Discussion

This case highlights the diagnostic challenges associated with papulonodular mucinosis, particularly in the absence of SLE. Alongside this, the patient’s clinical presentation evolved over several years, requiring multiple biopsies, extensive laboratory investigations, and medication trials to reach a diagnosis. Although PNM has been reported in the absence of systemic findings of SLE,^6,8^ or the first presenting feature of SLE,^8,9^ existing literature primarily focuses on patients with PNM that have an established SLE diagnosis. This complicates the diagnostic process when SLE and ANA positivity is absent. Additionally, PNM may be the sole cutaneous manifestation of LE for an extended period.^6^ The patient’s fluctuating symptoms and the clinical and histologic similarities to other diffuse papulonodular lesions underscores the importance of considering clinical history, physical examination findings, laboratory tests, and histopathology for accurate diagnosis.

Clinicians should be aware of the need for comprehensive evaluations and consider PNM in patients with persistent dermal nodules and papules, even in the absence of SLE. This case emphasizes the importance of considering a broad differential diagnosis and maintaining a reasonable index of suspicion for PNM in patients with chronic cutaneous mucinosis, regardless of SLE status. Further research and case documentation are essential to better understand the etiology and management of this rare condition. Due to its close association with SLE, patients suspected of having papulonodular mucinosis should be monitored closely for evidence of systemic involvement.

## References

[1] Rongioletti F . Chapter 46: Mucinoses. In: Bolognia J Schaffer JV Cerroni L (eds.) Dermatology. Vol 1. 5th ed. London: Elsevier, 2017, pp.753–764.

[2] Hansen E Pettit C Chung CG , et al. Papulonodular mucinosis with features of discoid lupus erythematosus. JAAD Case Rep 2024; 45: 59–61. 38389858 10.1016/j.jdcr.2023.12.022 PMC10882011

[3] Werth V Newman S . Cutaneous lupus mucinosis (papulonodular mucinosis, papular and nodular mucinosis of lupus, papular and nodular mucinosis of Gold). Dermatology Advisor. https://www.dermatologyadvisor.com/home/decision-support-in-medicine/dermatology/cutaneous-lupus-mucinosis-papulonodular-mucinosis-papular-and-nodular-mucinosis-of-lupus-papular-and-nodular-mucinosis-of-gold/ (published 13 March 2019, accessed 8 August 2024).

[4] Ortiz VG Krishnan RS Chen LL , et al. Papulonodular mucinosis in systemic lupus erythematosus. Dermatol Online J 2004; 10(2): 16. 15530306

[5] Lee WJ Park GH Chang SE , et al. Papular mucinosis associated with systemic lupus erythematosus. Ann Dermatol 2008; 20(4): 233. 27303200 10.5021/ad.2008.20.4.233 PMC4903987

[6] Sonntag M Lehmann P Megahed M , et al. Papulonodular mucinosis associated with subacute cutaneous lupus erythematosus. Dermatology 2003; 206(4): 326–329. 12771474 10.1159/000069945

[7] Ramamurthi A Bicknell L McCarty M . Hyaluronidase in the treatment of papular dermal mucinosis: first case reported in North America. J Surg Dermatol 2016; 1(2): 61–65.

[8] Gouveia AI Sousa M Osório-Valente R , et al. Papulonodular mucinosis: an unusual presenting feature of systemic lupus erythematosus. Int J Dermatol 2016; 55(11): e607–e608. 10.1111/ijd.13348 27337059

[9] Esteves M Gomes N Nogueira A , et al. Papulonodular mucinosis in the setting of systemic lupus erythematosus—a diagnostic conundrum. Int J Dermatol 2020; 59(12): e447–e449. 10.1111/ijd.15171 32857387

